# Bacterial fermentation platform for producing artificial aromatic amines

**DOI:** 10.1038/srep25764

**Published:** 2016-05-11

**Authors:** Shunsuke Masuo, Shengmin Zhou, Tatsuo Kaneko, Naoki Takaya

**Affiliations:** 1 Faculty of Life and Environmental Sciences, University of Tsukuba, Tsukuba, Ibaraki 305-8572, Japan; 2 School of Materials Science, Japan Advanced Institute of Science and Technology, 1-1 Asahidai, Nomi, Ishikawa, 923-1292 Japan

## Abstract

Aromatic amines containing an aminobenzene or an aniline moiety comprise versatile natural and artificial compounds including bioactive molecules and resources for advanced materials. However, a bio-production platform has not been implemented. Here we constructed a bacterial platform for *para*-substituted aminobenzene relatives of aromatic amines *via* enzymes in an alternate shikimate pathway predicted in a *Pseudomonad* bacterium. Optimization of the metabolic pathway in *Escherichia coli* cells converted biomass glucose to 4-aminophenylalanine with high efficiency (4.4 g L^−1^ in fed-batch cultivation). We designed and produced artificial pathways that mimicked the fungal Ehrlich pathway in *E. coli* and converted 4-aminophenylalanine into 4-aminophenylethanol and 4-aminophenylacetate at 90% molar yields. Combining these conversion systems or fungal phenylalanine decarboxylases, the 4-aminophenylalanine-producing platform fermented glucose to 4-aminophenylethanol, 4-aminophenylacetate, and 4-phenylethylamine. This original bacterial platform for producing artificial aromatic amines highlights their potential as heteroatoms containing bio-based materials that can replace those derived from petroleum.

The production of bulk materials and fuels from renewable resources is prerequisite for constructing sustainable low-carbon societies that can overcome global environmental issues and limited fossil fuel resources. Aromatic amines that are characterized by an amino-substituted benzene (aniline) moiety (referred to hereinafter as AA) serve as resources from which to develop dyes, rubbers, plastics and conductive polymers^1^,^2^, and they are important in a broad range of industries. Most living organisms produce the AA, 4-aminobenzoic acid, as a biosynthetic precursor of folate, which is an essential cofactor that is also a dietary supplement^3^. Some AA are intermediates of antibacterial chloramphenicol, pristinamycin^4^,^5^ and other drugs, and developing a repertoire of AA is important from a pharmaceutical standpoint. Due to such substantial demand, various commercial AA have been synthesized by petroleum chemistry, whereas none has been derived from biomass, which limits the molecular design of practical bio-derived products based on AA.

Aromatic amines are significant in the production of advanced polymer materials including functional and/or high-performance plastics. The amine group and the aromatic moiety of AA induce nucleophilic reactivity and excellent thermomechanical performance, respectively^6^. Aromatic amines are polycondensed with carbonyl compounds to generate aromatic polyamides, polyimides, polyazoles, polyurea and polyazomethines. When polycondensed with aromatic acids, AA generate super-engineering plastics with extremely high thermomechanical properties^7^,^8^. These include poly(*p*-phenylene terephthalamide (Kevlar^TM^) and poly(4,4′-oxydiphenylene pyromellitimide) (Kapton^TM^) that serve as thermostable materials in fabric for body armor and other flame-retardant materials, fiber-reinforced plastics for electronic devices, vehicle bodies and anti-pressure cylinders^7^,^8^,^9^. The applications of super-engineered plastics are diversifying, and this is increasing the annual global production of AA-derived plastics to around 100,000 tons. Global production of aromatic polyamides accounts for several hundreds of millions of US dollars, which indicates the size of the contribution of AA to both the economy and society.

The production of aromatic polyamides and polyimides requires aromatic carboxylic acids and/or AA as building blocks. Whereas aromatic carboxylic acids (such as terephthalic acid) have been derived from biomass^10^,^11^, AA have not, and this has precluded the development of fully bio-oriented aromatic polyamides and polyimides. Our recent microbial production of the non-natural AA, 4-aminocinnamic acid (4ACA), from 4-aminophenylalanine (4APhe), which is an intermediate of the chloramphenicol and pristinamycin biosynthesis pathway^4^,^5^, followed by synthesis of ultra-high-performance polyimide is the exception^12^. Not only 4ACA, but also other AA derived from biomass would serve as innovative monomers for synthesizing bio-AA plastics, and their environmental impact should be enormous, considering that they would replace polyamides and polyimides derived in bulk from petroleum.

Only a few natural pathways of AA biosynthesis have been documented. This causes considerable difficulty when trying to custom-design pathways to synthesize various AA. The 4ACA production system ferments 4APhe *via* antibiotic-synthesizing enzymes produced by recombinant *Escherichia coli*^13^, and then a bacterium producing phenylalanine ammonia lyase converts 4APhe to 4ACA (Fig. 1)^12^. This study identified a novel 4APhe synthetic gene cluster from *Pseudomonas fluorescens*, which in accordance with the optimization of host *E. coli* metabolism enables 35-fold more 4APhe production than that generated in a previous study that used antibiotic-synthesizing enzymes from *Streptomyces* bacteria (0.13 g L^−1^)^13^. Recombinant *E. coli* strains producing sets of phenylalanine-catabolic enzymes fermented glucose to a series of artificial 4-amino-substituted AA including 4-aminophenylacetate (4APAA), 4-aminophenylacetaldehyde, 4-aminophenylethanol (4APE), and 4-aminophenylethylamine (4APEA) (Fig. 1). Bio-aromatic polyamide and polyimides can be developed from all of these compounds. The platform for synthesizing bio-derived AA constructed in the present study increases the potential for producing a range of bio-based AA materials for bio-polymers and other applications.

## Results

### Novel *P. fluorescens papABC* genes optimize 4APhe production

We and another group expressed the *Streptomyces venezuelae* and *S. pristinaespiralis* genes encoding 4-amino-4-deoxychorismate synthase (PapA), 4-amino-4-deoxychorismate mutase (PapB), and 4-amino-4-deoxyprephenate dehydrogenase (PapC) in recombinant *E. coli* and synthesized 4APhe^13^. These enzymes convert cellular chorismate to 4-aminophenylpyruvate^4^,^5^, which endogenous aminotransferases in *E*. *coli* subsequently convert to 4APhe^13^. Homology searches identified similar amino acid sequences to *S. venezuelae* PapA, PapB and PapC in predicted proteins of *P. fluorescens* SBW25 (Fig. 2a) encoded by PFLU_1771 (*pfpapA*), PFLU_1772 (*pfpapB*) and PFLU_1770 (*pfpapC*), and their entire amino acid sequences have 44%, 28% and 34% similarity, respectively (Fig. 2a, Supplementary Fig. 1). These genes are clustered in the *P. fluorescens* SBW25 genome like their *Streptomyces* counterparts^4^,^5^, where they might constitute an operon. We constructed an artificial operon comprising the T7lac gene promoter followed by *pfpapB*, *pfpapA*, and *pfpapC* in that order (plasmid 1 in Fig. 2a), and introduced it into *E. coli* NST37(DE3)/Δ*pheLA*. The strain lacks a *pheLA* locus in the leader peptide for *pheA* expression (*pheL*) and chorismate mutase (*pheA*) that competes with the substrate chorismate with PapA. The strain harboring the operon generated 0.43 g L^−1^ 4APhe in minimal medium with glucose whereas all strains expressing only *pfpapA*, *pfpapAB* (Fig. 2b), *pfpapBC*, or the set of *pfpapA* and *pfpapC* (Supplementary Fig. 2) did not. These results indicated that *pfpapABC* participates in 4APhe synthesis.

We analyzed the ability of a series of *pfpapABC* expression plasmids to produce 4APhe. The appropriate genes were introduced into pET-duet1, pRSF-duet1 and pCDF-duet1, which were maintained as ~40, 20~40, and >100 copies/cell, respectively^14^, and expressed under the T7lac promoter (Fig. 2a). The strains harboring sets of pET-*pfpapA* and pCDF-*pfpapB*C, and pCDF-*pfpapA* and pET-*pfpapBC* (plasmids 3, 8 and 5, 6 in Fig. 2c, respectively) produced the most 4APhe, up to 0.53 g L^−1^. We selected NST37(DE3)/Δ*pheLA* harboring pET-*pfpapA* and pCDF-*pfpapB*C (NDP strain), which produced 4APhe, and optimized its 4APhe production.

### Metabolic engineering *E. coli* for efficient AA production

**Figure 1:**
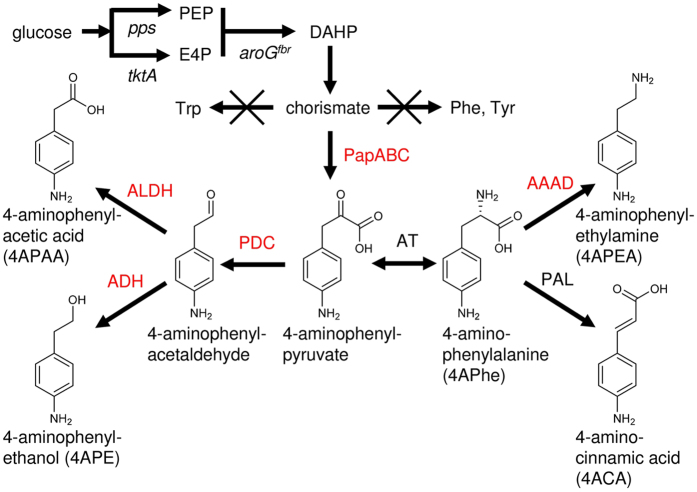
Artificial pathways producing aromatic amines. AAAD, aromatic amino acid decarboxylase; ADH, aldehyde dehydrogenase; ALDH, alcohol dehydrogenase; AT, aminotransferase; PAL, phenylalanine ammonia lyase; PapA, 4-amino-4-deoxychorismate synthase; PapB, 4-amino-4-deoxychorismate mutase; PapC, 4-amino-4-deoxyprephenate dehydrogenase; PDC, phenylpyruvate decarboxylase. Host cell genes encoded *aroG4*^*fbr*^, feedback resistant isozyme of DAHP synthase; *pps*, PEP synthase; *tktA*, transketolase.

**Figure 2:**
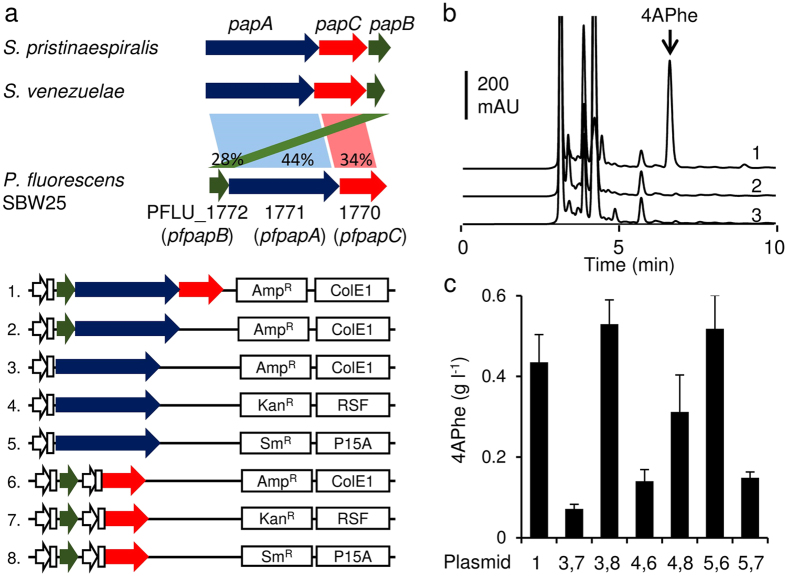
Identification and use of *pfpapABC* for 4APhe production. (**a**) Comparison of putative *pfpapABC* gene cluster of *P. fluorescens* SBW25 and *Streptomyces papABC* (upper panel). Plasmid organization for *pfpapBAC* expression (lower panel). (**b**) HPLC chromatograms of culture supernatant of *E. coli* after culture in 100 mL of fermentation medium in 500-mL flasks at 30 °C for 30 h. Numbers of introduced plasmids (panel **a**) are shown on traces. (**c**) Concentration of 4APhe in culture supernatant of *E. coli* harboring combinations of plasmids numbered as in (panel **a**). Error bars indicate standard deviation (*n* = 3).

**Figure 3:**
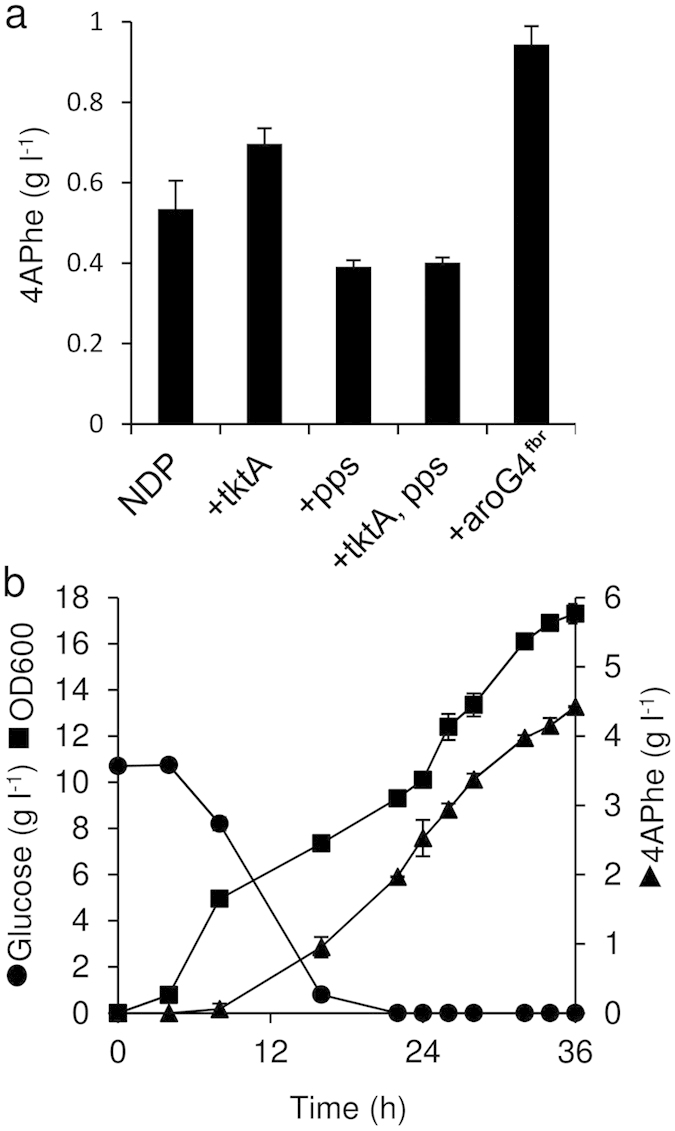
Metabolic engineering of *E. coli* to optimize 4APhe production. (**a**) 4APhe production by *E. coli* NDP expressing *pfpapABC* cultured in 100 mL of fermentation medium in 500-mL flasks at 30 °C for 30 h. Ectopically supplemented genes are shown below. Data are means of three experiments. Error bars indicate standard deviation. (**b**) Fed-batch cultivation of *E. coli* NDPG strain expressing *pfpapABC* and *aroG4*^*fbr*^. Fermentation medium contained more tryptone and yeast extract (5 and 2.5 g L^−1^, respectively). Glucose was fed to maintain concentration <1 g L^−1^. Error bars indicate standard deviation (*n* = 3).

We overexpressed the transketolase gene (*tktA*) under the control of the T7lac gene promoter in the NDP strain and found that it produced 1.2-fold more 4APhe than the parent strain (Fig. 3a), which is consistent with reports that its overexpression increases the availability of erythrose 4-phosphate (E4P) for 3-deoxy-D-arabinoheptulosonate 7-phosphate (DAHP) synthase, the rate-limiting enzyme of chorismate production, and hence cellular chorismate (Fig. 1)^15^,^16^ that is a substrate of the PapABC pathway. The enzyme phosphoenolpyruvate (PEP) synthase is used to synthesize cellular PEP that is another substrate of shikimate pathway besides E4P. Overexpression of the PEP synthase gene (*pps*) and both *tktA* and *pps* did not increase 4APhe production (Fig. 3a), indicating that *pps* decreased 4APhe production under our culture conditions. The PEP-dependent carbohydrate: phosphotransferase system (PTS) is a carbohydrate uptake process in *E. coli* where it is the major consumer of intracellular PEP^17^. Aiming to eliminate PEP consumption by PTS during cellular glucose uptake, we disrupted the *ptsHI-crr* genes that encode the cytoplasmic components of the system. Introducing pET-*pfpapA*/pCDF-*pfpapB*C into the gene disruptant resulted in lower 4APhe production compared with the corresponding non-disrupted strain (Supplementary Table 3), probably due to a decreased rate of glucose uptake.

We overexpressed *aroG* that encodes DAHP synthase to provide more chorismate for 4APhe production (Fig. 1). We used the *aroG4*^*fbr*^ gene that encodes a feedback inhibition-resistant (fbr) isozyme of DAHP synthase^18^. The NDPG strain overexpressing *aroG4*^*fbr*^ in the NDP strain produced 1.8-fold more 4APhe than the NDP strain in flask cultures (Fig. 3a) and this became more obvious when nitrogen sources in the culture medium were increased (Supplementary Table 4). Therefore, the following tests included bacteria cultured under these conditions. Overexpressing *tktA* and *pps* did not increase 4APhe production by the NDPG strain (Supplementary Table 4), whereas it increased the production of phenylalanine by a strain sharing the same *aroG*^*fbr*^ background^15^. These findings indicate that the amount of DAHP synthase limits bacterial 4APhe production under these conditions. We fed-batch cultured NDPG in a jar fermenter and optimized the conditions for 4APhe production. The glucose concentration decreased to <1 g L^−1^ after 16 h of batch culture and was maintained at this level thereafter to avoid repressing T7lac gene promoter activity^19^ and the metabolic overflow of glucose to acetate that inhibits *E. coli* fermentation^20^. Fed batch-cultured NDPG generated 4.4 g L^−1^ 4APhe with a production yield of 17% (*vs.* glucose) (Fig. 3b), which represents 62% of the theoretical yield of phenylalanine by *E. coli*^21^.

### Production of 4APE and 4APAA

**Figure 4:**
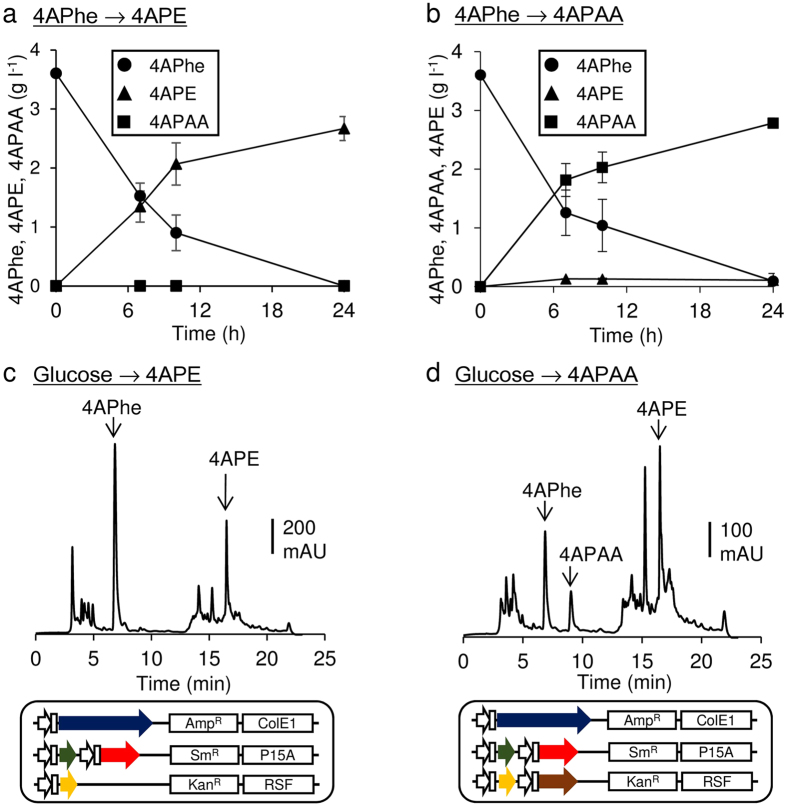
Production of 4APE and 4APAA. (**a,b**) Bioconversion of 4APhe to 4APE and 4APAA by *E. coli* BL21 Star (DE3) harboring either pRSF-aro10 (**a**) or pRSF-aro10ald2 (**b**). After incubation with 0.1 mM IPTG, cells were washed twice and incubated in reaction buffer containing 3.6 g L^−1^ 4APhe. Error bars indicate standard deviation (*n* = 3). (**c, d**) HPLC chromatogram of culture supernatant of NDPG harboring pRSF-aro10 (**c**) and pRSF-aro10ald2 (**d**). Cells were shaken in fermentation medium containing 5 g L^−1^ tryptone, 2.5 g L^−1^ yeast extract and 10 g L^−1^ glucose in flasks at 30 °C, induced with 0.1 mM IPTG, and cultured for 36 h.

The fungal Ehrlich pathway deaminates phenylalanine to phenylpyruvate, which phenylpyruvate decarboxylase (PDC) then converts to phenylacetaldehyde. Alcohol dehydrogenase (ADH) and aldehyde dehydrogenase (ALDH) reduce and oxidize phenylacetaldehyde to 2-phenylethanol and to phenylacetic acid, respectively^22^. Artificial pathways mimicking the Ehrlich pathway were designed to produce 4APE and 4APAA (Fig. 1) *via* pathway enzymes, although a 4-amino-substituted AA has never been assessed as an Ehrlich pathway intermediate. We constructed recombinant *E. coli* BL21 Star (DE3) producing *ARO10* encoding PDC from the fungus *S. cerevisiae*^22^, and examined the bioconversion of 4APhe in resting cells. The reaction consumed 1.8 g L^1^ (10 mM) of 4APhe to generate 1.2 g L^−1^ of 4APE with a 90% molar yield (Table 1), implying that PDC efficiently produced 4-aminophenylacetaldehyde, which endogenous *E. coli* ADH reduced to 4APE. The resting *E. coli* BL21 Star (DE3) cells producing both *ARO10* and ALDH encoding *ALD3*^22^ converted 1.8 g L^−1^ of 4APhe to 0.9 g L^−1^ of 4APAA with a 63% molar yield (Table 1). The strain produced 0.4 g L^−1^ 4APE as a byproduct, indicating that the dehydrogenation of 4-aminophenylacetaldehyde limits 4APAA production. Replacing *ALD3* with *E. coli padA*^23^ and *S. cerevisiae ALD2*^22^ in the bacterium expressing *ARO10* and *ALD3* increased the yields of 4APAA to 85% and 90%, respectively (Table 1). We also combined PDC, *Pichia pastoris PpARO10*^24^ or *Aspergillus oryzae ppdA*^25^ with *ALD3*, but they produced less 4APAA than those expressing *ARO10* and *ALD3* (Table 1). These results indicated that the heterologous expression of *ARO10* alone and of the *ARO10* and *ALD2* set converted 4APhe to 4APE and APAA the most efficiently. Figure 4a,b shows the time-dependent bioconversion of 4APhe (3.6 g L^−1^) in the optimized systems that produced 2.8 g L^−1^ 4APE and 2.7 g L^−1^ 4APAA.

We expressed *ARO10* or both *ARO10* and *ALD2* in the NDPG strain expressing *pfpapABC*. The resulting NDPGA and NDPGAA strains cultured in fermentation medium produced 4APE, and both 4APAA and 4APE, respectively. Production increased linearly until reaching a maximum of 0.24 g L^−1^ 4APE (Fig. 4c), 0.12 g L^−1^ 4APAA and 0.19 g L^−1^ 4APE (Fig. 4d) at 36 h. These results showed that the cultures fermented glucose to 4APE and 4APAA. Both strains accumulated less fermentation products than the intermediate, 4APhe (0.4–1.2 g L^−1^) (Fig. 4c,d). Further optimization of growth parameters or genetic modification could hasten conversion of the intermediate and produce more 4APE and 4APAA. Fermentation by the NDPGAA generated 4APE as a byproduct (Fig. 4d), while bioconversion by *E. coli* BL21 Star (DE3) expressing *ARO10* and *ALD3* resulted in little 4APE production (Fig. 4b), indicating metabolic differences between the strains. These results demonstrated that the new bacterial platform can efficiently produce biomass-derived AA.

### Production of 4APEA

**Figure 5:**
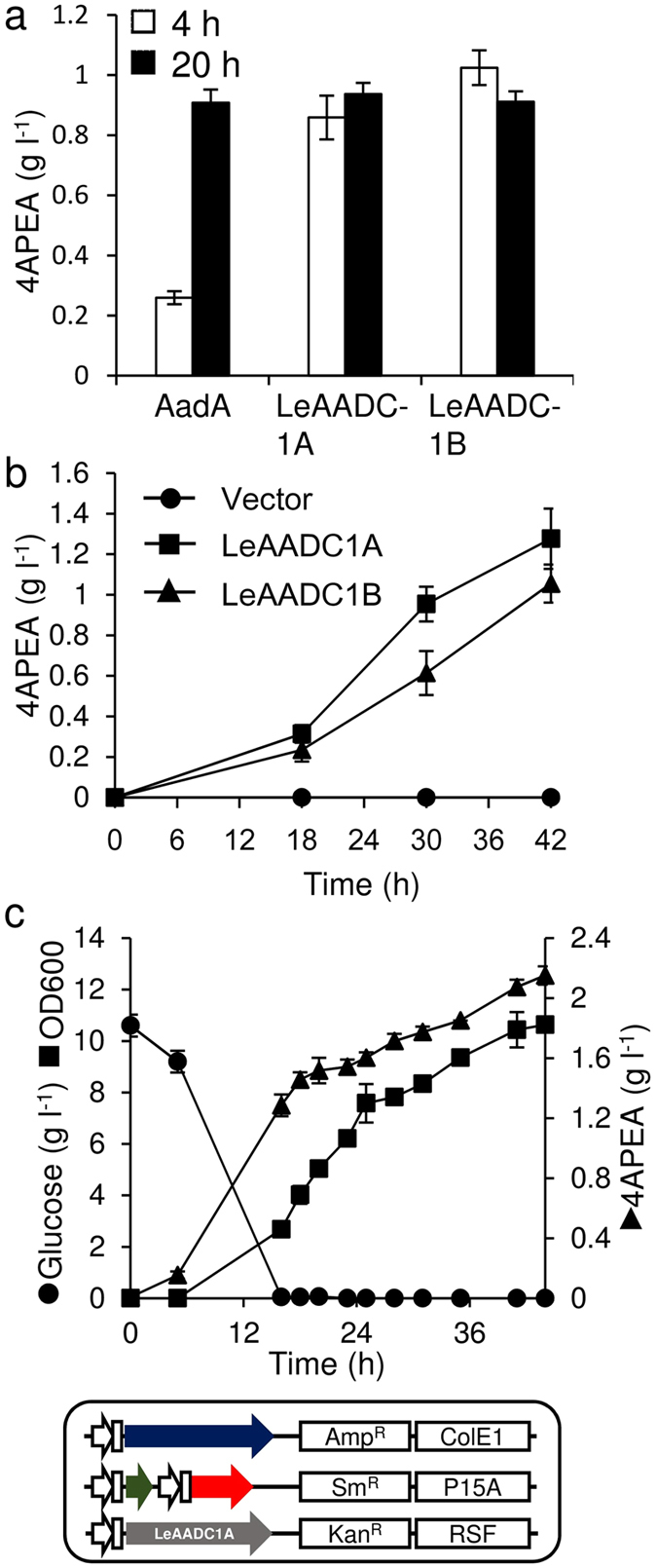
Production of 4APEA. (**a**) Bioconversion of 4APhe to APEA by *E. coli* harboring pRSF-aadA, pRSF-leaadc1A, and pRSF-leaadc1B. Cells grown in flasks of LB medium containing 0.1 mM IPTG at 30 °C were incubated with 1.8 g L^−1^ 4APhe. Error bars indicate standard deviation (*n* = 3). (**b**) Fermentation of 4APEA by NDPG harboring pRSF-leaadc1A or pRSF-leaadc1B. Cells were grown at 30 °C in flasks of fermentation medium containing 5 g L^−1^ tryptone and 2.5 g L^−1^ yeast extract and 0.1 mM IPTG. Error bars indicate standard deviation (*n* = 3). (**c**) Fed-batch culture of NDPG harboring pRSF-leaadc1A in jar fermenters containing 0.5 L of fermentation medium containing 5 g L^−1^ tryptone and 2.5 g L^−1^ yeast extract at 30 °C. Adding ammonium maintained pH at 7.0. Glucose (0.8 g L^−1^ h^−1^) was fed at 16 h after starting culture. Error bars indicate standard deviation (*n* = 3).

We examined the reaction of aromatic L-amino acid decarboxylase, which decarboxylates phenylalanine to phenylethylamine and carbon dioxide, against 4APhe (Fig. 1). We constructed recombinant *E. coli* BL21 Star (DE3) overexpressing *A. oryzae aadA*^25^ and either of the *Solanum lycopersicum LeAADC1A* and *LeAADC1B*^26^ genes, both of which encode aromatic L-amino acid decarboxylases. Incubating these cells with 1.8 g L^−1^ (10 mM) 4APhe efficiently converted 4APhe to 4APEA with 65% to 75% molar yield (*vs.* 4APhe) (Fig. 5a). The strain expressing either *LeAADC1A* or *LeAADC1B* produced 4APEA within 4 h. We introduced these genes into the NDPG strain, and cultured it in fermentation medium containing 1% glucose. After 42 h, 1.3-fold more 4APEA was accumulated by the strain expressing *LeAADC1A* than *LeAADC1B* (Fig. 5b), indicating that both strains harboring *LeAADC1A* converted glucose biomass to 4APEA more efficiently. Fed-batch cultures of the strain expressing *LeAADC1A* in a jar fermenter generated 1.8 g L^−1^ 4APEA after 44 h (Fig. 5c). The production yield (*vs.* glucose) of 4APEA was 5.6%, which was below its theoretical yield (23%) calculated from that for phenylalanine (27.5%)^21^.

## Discussion

The aim of constructing microbial platforms to produce various groups of chemicals is to develop specific pharmaceuticals and biomass-derived materials that can substitute for petroleum chemicals. Most current targets for petroleum-based chemicals comprise aliphatic molecules, whereas only aromatic amino acids are fermented using glucose biomass as a carbon donor. Glucose is bio-converted into shikimate pathway intermediates^27^ and deoxy-*scyllo*-inosose^28^, both of which are chemically derivatized to aromatic compounds such as catechol and its related compounds. Bio-derived terephthalic acid has recently been synthesized using biomass-derived isobutanol or 5-hydroxymethylfurfral^11^. These aromatic compounds were generated by combining microbial, with chemical reactions. However, we explored biological production systems that do not require chemical processes to produce artificial aromatic chemicals, especially those with a substituted nitrogen atom. This is the first bacterial platform for the direct fermentative production of a series of AA, and represents a promising environmentally-friendly alternative for synthesizing AA building blocks that are applicable to numerous technologies.

This study integrated metabolic engineering strategies to produce AA. Firstly, established strategies for high-level fermentation of phenylalanine as well as perturbed metabolic flow of chorismate to phenylalanine improved chorismate synthesis by *E. coli.* Secondly, introducing exogenous *papABC* genes integrated the intrinsic *E. coli* pathway for chorismate synthesis with the pathway generating aminophenylpyruvate from chorismate, and fermented glucose to 4APhe (Fig. 1). We found that *papABC* is indispensable for AA production and that the *P. fluorescens papABC* identified herein and which originated from a proteobacterium similar to *E. coli* optimized production efficiency. Thirdly, we searched bacteria, fungi and plants for enzymes that produce shikimate pathway derivatives, and used them to produce 4APEA, 4APE, and 4APAA from a sugar biomass. The production rates are sufficient to generate industrial quantities of pharmaceutical intermediates. Fed-batch fermentation produced optimal amounts of 4APEA (1.8 g L^−1^), which will be improved by applying traditional strategies such as continuous culture and genetically engineering host *E. coli* metabolism, and 4APEA generated in this manner will have potential for bio-material production after a purification process is established.

The platform produced biomass-derived aromatics containing an aniline structure with a substitution at the *para*-position *via* a C2 chain linked to a functional (amine or carbonyl) group. Such structures have unique molecular properties; 4APEA is a diamine connected to rigid aromatic (C6) moiety and flexible C2 chain. This and structurally similar bipartite diamines are polymerized with both aliphatic and aromatic dicarboxylic acids to generate thermostable polyamides with a considerably high glass-transition temperature^29^. Polyamide generated from 4APEA and isophthalic acid has high thermal stability (*T*_d10_ = 360 °C)^30^. Polycondensation with long flexible aliphatic acids generates 4APEA with liquid crystallinity. Furthermore, 4APAA is a C6:C2 compound, and an aromatic amino acid. The related 4-aminobenzoic acid (C6:C1) is homopolymerized to form poly(*p*-benzamide) with high thermomechanical properties but its poor solubility and the absence of a melting temperature causes difficulties with processing them into plastics. Copolymerizing 4-aminobenzoic acid with 4APAA increases flexibility through the addition of alkyl chains connected to the aromatic rings of poly(*p*-benzamide)^31^. This molecular design improves processability into heat-resistant filaments, fibers and films^31^. Thus, the present findings should impact the development of novel bio-based aromatic materials.

We developed an AA production platform based on a shikimate pathway variant for synthesizing 4APhe (Fig. 1). Although only a small variety of compounds are derived from this pathway in nature, the platform produced the artificial 4-amino-substituted compounds 4APAA, 4APE and 4APAE in combination with the enzymes for synthesizing their phenylalanine derivatives. Natural derivatives of phenylalanine and its synthetic intermediates are more diverse than 4APhe-related compounds, and include cinnamic acid, cinnamyl alcohol, cinnamaldehyde, homogentisic acid, phenyllactic acid, mandelic acid, styrene, benzoic acid and other aromatic compounds^32^. We use phenylalanine-deaminating phenylalanine ammonia lyase (Fig. 1) in the bioconversion of 4APhe to 4ACA^11^. Other enzymes that biosynthesize phenylalanine derivatives are potential catalysts in the generation of artificial AA, but their synthesis awaits investigation. The constructed platform for AA synthesis has the potential to produce bio-derived non-natural AA.

## Methods

### Bacterial strains and reagents

Supplementary Table 1 lists the strains used in this study. *Escherichia coli* NST37 (ATCC31882) was lysogenized using λDE3 Lysogenization kits (Novagen, Madison, WI, USA) to generate NST37 (DE3). We obtained 4APhe from Sigma Aldrich (St. Louis, MO, USA) and 4ACA, 4APE and 4APEA from Tokyo Chemical Industry (Tokyo, Japan). Plasmids were constructed using PrimeSTAR HS DNA polymerase and restriction enzymes (Takara Bio Inc., Shiga, Japan).

### Constructing plasmids for 4APhe production

Supplementary Table 2 lists the primers used in this study. Artificially synthesized, codon-optimized *pfpapA*, *pfpapB* and *pfpapC* (GenScript, NJ, USA) (accession numbers; KU199222, KU199223, and KU199224) were cloned into pUC57 to generate pUC-pfpapA, pUC-pfpapB and pUC-pfpapC, respectively. DNA fragments for *pfpapA*, *pfpapB* and *pfpapC* were amplified by PCR using these plasmids and the respective primers sets, PFAF/PFAR, PFBF/PFBR and PFCF/PFCR, and then fused by PCR using the primers PFBF and PFCR to generate *pfpapBAC* fragments. These fragments were digested with *Nde*I and *Xho*I and cloned into pET22b to generate pET-pfpapBAC. Fragments of pfpapBA were amplified by PCR using PFBF and PFAR2 primers and pET-pfpapBAC, digested with *Nde*I and *Xho*I, and cloned into pET22b to generate pET-pfpapBA. We digested pUC-pfpapA with *Nde*I and *Xho*I, and then purified *pfpapA* fragments were cloned into pET-duet1, pCDF-duet1 and pRSF-duet-1 (Novagen) that were digested with the same enzymes to generate pET-pfpapA, pCDF-pfpapA and pRSF-pfpapA. We digested pUC-pfpapB with *Nco*I and *Not*I, and inserted them into the same vectors to generate pET-pfpapB, pCDF-pfpapB and pRSF-papB. We digested pUC-pfpapC with *Nde*I and *Xho*I and cloned *pfpapC* fragments into these plasmids to generate pET-pfpapBC, pCDF-pfpapBC and pRSF-papBC.

### Genetic engineering of intrinsic *E. coli* metabolic pathways

The synthesized DNA fragment *aroG4*^*fbr*^ 18 was cloned into pACYC184 that was digested with *Eco*RV and *Hi*ndIII and treated with the Klenow fragment to create pACYC-aroG4. The *tktA* and *pps* genes were amplified by PCR using *E. coli* MG1655 total DNA and primers, digested with *Nco*I and *Bam*HI, and *Nde*I and *Xho*I, and cloned into pRSFduet-1 to generate pRSF-tktA and pRSF-pps, respectively. The *pps* gene was digested with *Nde*I and *Xho*I and also cloned into pRSF-tktA to generate pRSF-tktApps.

Knock-out cassettes generated by PCR using the primers DpheLAF and DpheLAR, and the Red/ET recombination system (Gene Bridges, Heidelberg, Germany) replaced the genomic *pheLA* locus of *E. coli* NST37(DE3) with the kanamycin resistance gene (Km^r^). The FLP/FRT recombination technique (Gene Bridges) deleted Km^r^ from the strain and generated *E. coli* NST37(DE3)/Δ*pheLA*. Diagnostic PCR proceeded using the primers C1 and C2. Likewise, the *ptsHI*-*crr* genes of NST37(DE3)/Δ*pheLA* were knocked-out using the cassette-generated primer pairs DptsHI-crrF and DptsHI-crrR to generate NST37(DE3)/Δ*pheLA*/Δ*ptsHI-crr*. Primer pairs C3 and C4 was used for diagnostic PCR.

### Constructing plasmids for production of other AA

The *ARO10* and *ALD3* genes were amplified by PCR using total DNA from *S. cerevisiae* and appropriate primers. The *ARO10* fragment was digested with *Nco*I and *Bam*HI and cloned into pRSFduet-1 to generate pRSF-aro10. The ALD3 fragment was digested with *Nde*I and *Xho*I and cloned into pRSFduet-1 and pRSF-aro10 to generate pRSF-ald3 and pRSF-aro10ald3, respectively. The *S. cerevisiae ALD2* and the *E. coli padA* genes were amplified using the appropriate primers, digested with *Nde*I and *Xho*I, and cloned into pRSF-aro10 to generate pRSF-aro10ald2 and pRSF-aro10padA. The PpARO10 (GenBank accession number, CCA40086.1) gene was amplified by PCR using *Pichia pastoris* total DNA and primers, digested with *Sac*I and *Not*I, and then cloned into pRSF-ald3 to generate pRSF-pparo10ald3. The *A. oryzae ppdA* was amplified using primers, digested with *Nco*I and *Hin*dIII, and cloned into pRSFduet-1 to generate pRSF-ppdA. The *Nde*I and *Xho*I fragment of *ALD3* was cloned into pRSF-ppdA to generate pRSF-ppdAald3.

*Solanum lycopersicum* LeAADC1A and LeAADC1B were amplified using its cDNA and the appropriate primers. The *A. oryzae aadA* gene was amplified using pET-aadA and the appropriate primers. These DNA fragments were digested with *Bam*HI and *Hin*dIII, and cloned into pRSFduet-1 to generate pRSF-leaadc1a, pRSF-leaadc1b and pRSF-aadA.

### Fermentation of 4APhe, 4APAA, 4APE and 4APEA

Cells were grown in the fermentation medium comprising 10 g glucose, 2 g tryptone, 1 g yeast extract, 6 g Na_2_HPO_4_, 3 g KH_2_PO_4_, 0.5 g NaCl, 2 g NH_4_Cl, 0.5 g MgSO_4_ 7H_2_O, 15 mg CaCl_2_, 20 mg tyrosine, 20 mg tryptophan, 20 mg phenylalanine, 50 mg thiamine-HCl and 1 mL of trace element solution/L^33^. The plasmids were maintained by adding 100 mg L^−1^ sodium ampicillin, 40 mg L^−1^ kanamycin sulfate and 35 mg L^−1^ chloramphenicol. Recombinant *E. coli* generated from NST37(DE3)/Δ*pheLA* was pre-cultured by rotary shaking at 300 rpm overnight in test tubes containing 5 mL of LB medium at 28 °C, and inoculated into 100 mL of fresh M9 medium in 500-mL conical flasks at 1:100 dilution. When the cells reached an optical density (OD) of 0.6 at 600 nm and 30 °C, 0.1 mM isopropyl-β-d-thiogalactoside (IPTG) was added. After 20–24 h of cultivation the glucose concentration reached <2 g L^−1^ and then 2 mL of 500 g L^−1^ glucose was added and the cells were further cultured in the flasks for 48 h. Fed-batch cultures were agitated at 30 °C and 550 rpm in a 1.0-L BMJ-01 fermenter (Biott, Tokyo, Japan) containing 0.5 L of fermentation medium supplemented with 5 g L^−1^ tryptone, 2.5 g L^−1^ yeast extract, 10 g L^−1^ (NH_4_)_2_SO_4_ and 10 g L^−1^ glucose. The culture was aerated at 0.6 L min^−1^. When the OD reached 0.6 at 600 nm, 0.1 mM IPTG was added. Peristaltic pumps fed the cultures with 500 g L^−1^ of glucose and 0.1 mM IPTG when the glucose concentration dipped below 1.5 g L^−1^. The pH was monitored using an electrode and maintained between 7.0 and 7.1 by adding 10% NH_4_OH.

### Bioconversion by resting cells

*Escherichia coli* BL21 Star (DE3) (Invitrogen, Carlsbad, CA, USA) harboring expression plasmids was rotary shaken at 300 rpm and 30 °C overnight in test tubes containing 3 mL of LB medium and inoculated into 500-mL conical flasks containing 100 mL LB medium at a 1:100 dilution. When the cells reached an OD of 0.6 at 600 nm and 30 °C, 0.1 mM IPTG was added. Cells harboring pRSF-aro10, pRSF-aro10ald3, pRSF-aro10ald2 or pRSF-aro10padA were further incubated at 30 °C for 6 h. The cells were collected by centrifugation at 5,000 × *g* for 10 min and washed with DCD buffer (100 mM KH_2_PO_4_ (pH 7.5), 1 mM MgSO_4_, 0.5 mM thiamine chloride). Cells harboring pRSF-leaadc1a, pRSF-leaadc1b or pRSF-aadA were further incubated at 16 °C for 12 h with 0.5 mM pyridoxal 5-phosphate, washed with AAD buffer (100 mM potassium phosphate, pH 7.5 and 0.5 mM pyridoxal 5-phosphate). The washed cells were incubated in 10 mL of DCD or AAD buffer containing the indicated amounts of 4APhe at 28 °C and shaken at 300 rpm for the indicated periods.

### Determination of metabolites

We determined yields of 4APhe, 4APA, 4APE and 4APEA by high-performance liquid chromatography (HPLC) using a 1200 infinity series (Agilent Technologies, Palo Alto, CA, USA) equipped with a 250 × 4.6-mm Purospher Star RP-18 end-capped column with a particle size of 5 μm (Millipore-Merck, Billerica, MA, USA). The initial mobile phase was solvent A:solvent B = 98:2 (solvent A, 20 mM potassium phosphate (pH 7.0); solvent B, methanol) and maintained for 7 min. The concentration of solvent B was increased to 50% for 5 min and then maintained at that ratio for another 5 min. The flow rate was 0.8 mL min^−1^, and absorption at 210 nm was monitored. Glucose concentrations were determined using glucose-CII test kit (Wako, Tokyo, Japan).

## Additional Information

**How to cite this article**: Masuo, S. *et al.* Bacterial fermentation platform for producing artificial aromatic amines. *Sci. Rep.* **6**, 25764; doi: 10.1038/srep25764 (2016).

## Supplementary Material

Supplementary Information

## Figures and Tables

**Table 1:** Production of 4APAA and 4APE from 4APhe with PDC and ALDH in *E. coli*.

PDC	ALDH	Product (g L^−1^)	
		4APAA	4APE
*ARO10*	–	<0.01	1.2 (0.90)
*ARO10*	*ALD3*	0.9 (0.63)	0.4 (0.28)
*ARO10*	*ALD2*	1.4 (0.90)	0.1 (0.09)
*ARO10*	*padA*	1.3 (0.85)	0.03 (0.01)
*PpPDC*	*ALD3*	<0.01	<0.01
*ppdA*	*ALD3*	0.4 (0.28)	0.1 (0.10)

*Escherichia coli* BL21 Star (DE3) with pRSF-based plasmid expressed PDC and ALDH genes. Washed cells were incubated in Tris-HCl (pH 8.5) containing 1.8 g L^−1^ 4APhe at 30 °C for 16 h. Products were measured by HPLC. Production yields (*vs.* 4APhe) are shown in parenthesis. Standard error is <10%.
