# Supplementary material for: Bacterial fermentation platform for producing artificial aromatic amines
Source: Sci Rep. 2016 May 11;6:25764. doi: 10.1038/srep25764 (PMC4863162; doi:10.1038/srep25764)

Bacterial fermentation platform for producing artificial aromatic amines

Shunsuke Masuo[1], Shengmin Zhou[1], Tatsuo Kaneko[2] & Naoki Takaya[1]

[1]Faculty of Life and Environmental Sciences, University of Tsukuba, Tsukuba, Ibaraki 305-8572, Japan. [2]School of Materials Science, Japan Advanced Institute of Science and Technology, 1-1 Asahidai, Nomi, Ishikawa, 923-1292 Japan.

Correspondence and requests for materials should be addressed to N.T.
Email: takaya.naoki.ge@u.tsukuba.ac.jp

Supplementary Table 1 | Strains used in this study.

E. coli strains	Relevant genotype	Reference
NST37	aroF^{fbr}, aroG4^{fbr}, tyrA, tyrR, pheAfbr, pheAo, trpE	ATCC31882
NDP1	NST37(DE3)/*ΔpheLA* harboring pET-pfpapBAC	This study
NDP2	NST37(DE3)/*ΔpheLA* harboring pET-pfpapBA	This study
NDP3	NST37(DE3)/*ΔpheLA* harboring pET-pfpapA	This study
NDP3,7	NST37(DE3)/*ΔpheLA* harboring pET-pfpapA/pRSF-	This study
	pfpapBC	
NDP	NST37(DE3)/*ΔpheLA* harboring pET-pfpapA/pCDF-	This study
NDP4,6	pfpapBC	This study
NDP4,8	NST37(DE3)/*ΔpheLA* harboring pRSF-pfpapA/pET-	This study
NDP5,6	pfpapBC	This study
NDP5,7	NST37(DE3)/*ΔpheLA* harboring pRSF-pfpapA/pCDF-	This study
NDPT	pfpapBC	This study
NDPP	NST37(DE3)/*ΔpheLA* harboring pCDF-pfpapA/pET-	This study
NDPTP	pfpapBC	This study
	NST37(DE3)/*ΔpheLA* harboring pCDF-pfpapA/pRSF-	
	pfpapBC	
	NDP harboring pRSF-tktA	
	NDP harboring pRSF-pps	
	NDP harboring pRSF-tktApps	
NDPG	NDP harboring pACYC-aroG4	This study
NDPGT	NDPG harboring pRSF-tktA	This study
NDPGTP	NDPG harboring pRSF-tktApps	This study
NDPGA	NDPG harboring pRSF-aro10	This study
NDPGAA	NDPG harboring pRSF-aro10ald2	This study
NDPGADa	NDPG harboring pRSF-leaadc1a	This study
NDPGADb	NDPG harboring pRSF-leaadc1b	This study
BL21 Star (DE3)	F- *omp*T *hsd*S_B (r_B^-, m_B^-) *gal dcm rne*131 (DE3)	This study
BA	BL21 Star (DE3) harboring pRSF-aro10	This study
BAA3	BL21 Star (DE3) harboring pRSF-aro10ald3	This study
BAA2	BL21 Star (DE3) harboring pRSF-aro10ald2	This study
BAP	BL21 Star (DE3) harboring pRSF-aro10padA	This study
BppAA3	BL21 Star (DE3) harboring pRSF-aro10ald3	This study
BaoAA3	BL21 Star (DE3) harboring pRSF-ppdAald3	This study
BADa	BL21 Star (DE3) harboring pRSF-leaadc1a	This study
BADb	BL21 Star (DE3) harboring pRSF-leaadc1b	This study
BADao	BL21 Star (DE3) harboring pRSF-aadA	This study

Supplementary Table 2 | List of primers.

Name	Nucleotide sequence 5’-3’	Used to generate
PFAF	5′-atgaaaattctgctgattgac-3′	*pfpapA*
PFAR	5′-tcatttttcactaccggcatag-3′	*pfpapA*
PFBF	5′-cgacacacatatgaatatgaccgaacaccgc-3′	*pfpapB*
PFBR	5′-gtcaatcagcagaattttcatgagcgttgaccctggttac-3′	*pfpapB*
PFCF	5′-ctatgccggtagtgaaaaatgaacacgaacacggtggtgg-3′	*pfpapC*
PFCR	5′-gcactcgagttagttcagcagggaaaacag-3′	*pfpapC*
PFAR2	5′-cgactcgagtcatttttcactaccggca-3′	*pfpapA*
TktF	5′-gcaccatgggctcctcacgtaaagagcttgc-3′	*E. coli tktA*
TktR	5′-gagggatccttacagcagttcttttgctttc3′	*E. coli tktA*
PpsF	5′-cgacacacatatgtccaacaatggctcgtc -3′	*E. coli pps*
PpsR	5′-cagctcgagttatttcttcagttcagccagg -3′	*E. coli pps*
AR10F	5′- gagccatggcacctgttacaattga -3′	*S. cerevisiae ARO10*
AR10R	5′- gacggatcctatttttatttcttttaagtgc -3′	*S. cerevisiae ARO10*
AL3F	5′- ccagagacatatgcctaccttgtatactga -3′	*S. cerevisiae ALD3*
AL3R	5′- ctgctcgagttatttatccaatgaaagatcc -3′	*S. cerevisiae ALD3*
PpAR10F	5′- gtgagctcatggccccagttccagatatagc -3′	*P. pastoris ARO10*
PpAR10R	5′- gacagcggccgcttaacctacgattttggctttg -3′	*P. pastoris ARO10*
PpdF	5′- cagccatggcgacagatatcgctacaagg -3′	*A. oryzae ppdA*
PpdR	5′- gacaagcttaaagggacttgttccgctcag -3′	*A. oryzae ppdA*
AL2F	5′-cgacacacatatgcctaccttgtatactga -3′	*S. cerevisiae ALD2*
AL2R	5′-cagctcgagttagttgtccaaagagaga-3′	*S. cerevisiae ALD2*
PadF	5′- cgagacacatatgacagagccgcatgtagc -3′	*E. coli padA*
PadR	5′- cagctcgagttaataccgtacacacaccg -3′	*E. coli padA*
ADC1AF	5′-gacggatccatgggtagtctctcacttgaaatgg -3′	Tomato *LeAADC1A*
ADC1AR	5′-gacaagcttctaaggacagatgtagtcaatc -3′	Tomato *LeAADC1A*
ADC1BF	5′-gacggatccatgggtagcctcacacttgaaatgg -3′	Tomato *LeAADC1B*
ADC1BR	5′-gacaagcttctaaggaccaatataatcaatc -3′	Tomato *LeAADC1B*
AADF	5′-gacggatccatggaccgcgaccagtttagagc -3′	*A. oryzae aadA*
AADR	5′-gacaagcttcaattcgaccgacctagcacc -3′	*A. oryzae aadA*
DpheLAF	5′-tgaaaacagtacgggtactgtactaaagtcacttaaggaaacaaacatggaagt<br>tcctattctctagaaagtataggaacttctggacagcaagcgaaccggaattgc -3′	*pheLA* disruption
DpheLAR	5′- gatgattcacatcatccggcaccttttcatcaggttggatcaacaggcacgaagt<br>tcctatactttctagagagaataggaacttctcagaagaactcgtcaagaaggcg -3′	*pheLA* disruption
C1	5′- gagtagtcctttatattgagtg -3′	diagnostic PCR for
C2	5′- cgaatgacaatcgccagtaat -3′	*pheLA* disruption
DptsHI-crrF	5′-aggctagactttagttccacaacactaaacctataagttggggaaatacagaag<br>ttcctattctctagaaagtataggaacttctggacagcaagcgaaccggaattgc-3′	*ptsHIcrr* disruption
DptsHI-crrR	5′-aagcataaaaaaatggcgccgatgggcgccatttttcactgcggcaagaagaa<br>gttcctatactttctagagaataggaacttctcagaagaactcgtcaagaagg -3′	*ptsHI-crr* disruption
C3	5′- aactggcgctaacaatacag -3′	diagnostic PCR for
C4	5′- tacacccagcagcatgagag -3′	*ptsHIcrr* disruption

Supplementary Table 3 | Effect of *ptsHI-crr* deletion and spontaneous Glucose$^+$ mutation upon 4APhe production.

**Strain**	**OD_{600}**	**Glucose consumption ($g\ L^{-1}\ h^{-1}$)**	**4APhe (g/L)**
**NST37(DE3)/*ΔpheLA*/pfpapABC/aroG4**	7.5	0.42	1.8
**NST37(DE3)/ *ΔpheLA*/*ΔptsHI-crr*/ pfpapABC/aroG4**	1.5	0.12	0.02
**NST37(DE3)/ *ΔpheLA*/*ΔptsHI-crr*/ pfpapABC/ aroG4/ Glucose$^+$**	7.5	0.29	0.35

Glucose$^+$ mutant was obtained as described by Biville, F. et al. (J. Gen. Microbiol. 137, 1775-82 (1991)).

Supplementary Table 4 | Culture media optimization for 4APhe production.

Strains were cultured in 500-mL conical flasks containing 100 mL of various M9-based media at 30°C for 24 h. Concentrations of 4APhe were measured as described in Materials and Methods.

Strain	Yeast extract ($g\ L^{-1}$)	Tryptone ($g\ L^{-1}$)	Ammonium chloride ($g\ L^{-1}$)	Ammonium sulfate ($g\ L^{-1}$)	4APhe ($g\ L^{-1}$)
**NDP**	1.0	2.0	2.0	-	0.3
	2.5	5.0	2.0	-	0.1
**NDPG**	1.0	2.0	2.0	-	0.7
	2.5	5.0	2.0	-	1.2
	2.5	5.0	4.0	-	1.7
	2.5	5.0	6.0	-	1.6
	2.5	5.0	10.0	-	1.3
	2.5	5.0	-	2.0	1.1
	2.5	5.0	-	4.0	1.8
	2.5	5.0	-	6.0	1.9
	2.5	5.0	-	10.0	1.9
**NDPG + tktA**	2.5	5.0	-	10.0	1.7
**NDPG + tktA, pps**	2.5	5.0	-	10.0	1.8

Figure legend for Supplementary figure 1

Supplementary figure 1 | Alignment of amino acid sequences encoded by *papA* (a), *papB* (b) and *papC* (c). The genes from *S. venezuelae* and *S. pristinae* are prefixed with “Sv” and “Sp”, respectively. Pflu1771, Pflu1772 and Pflu1770 are *P. fuluoresens papA*, *papB*, and *papC*. Conserved amino acids are highlighted.

Supplementary figure 2 | HPLC chromatograms of culture supernatant of *E. coli* after culture in 100 mL of fermentation medium in 500-mL flasks at 30°C for 36 h. Introduced genes are shown on traces.

Supplementary Fig. 1

a

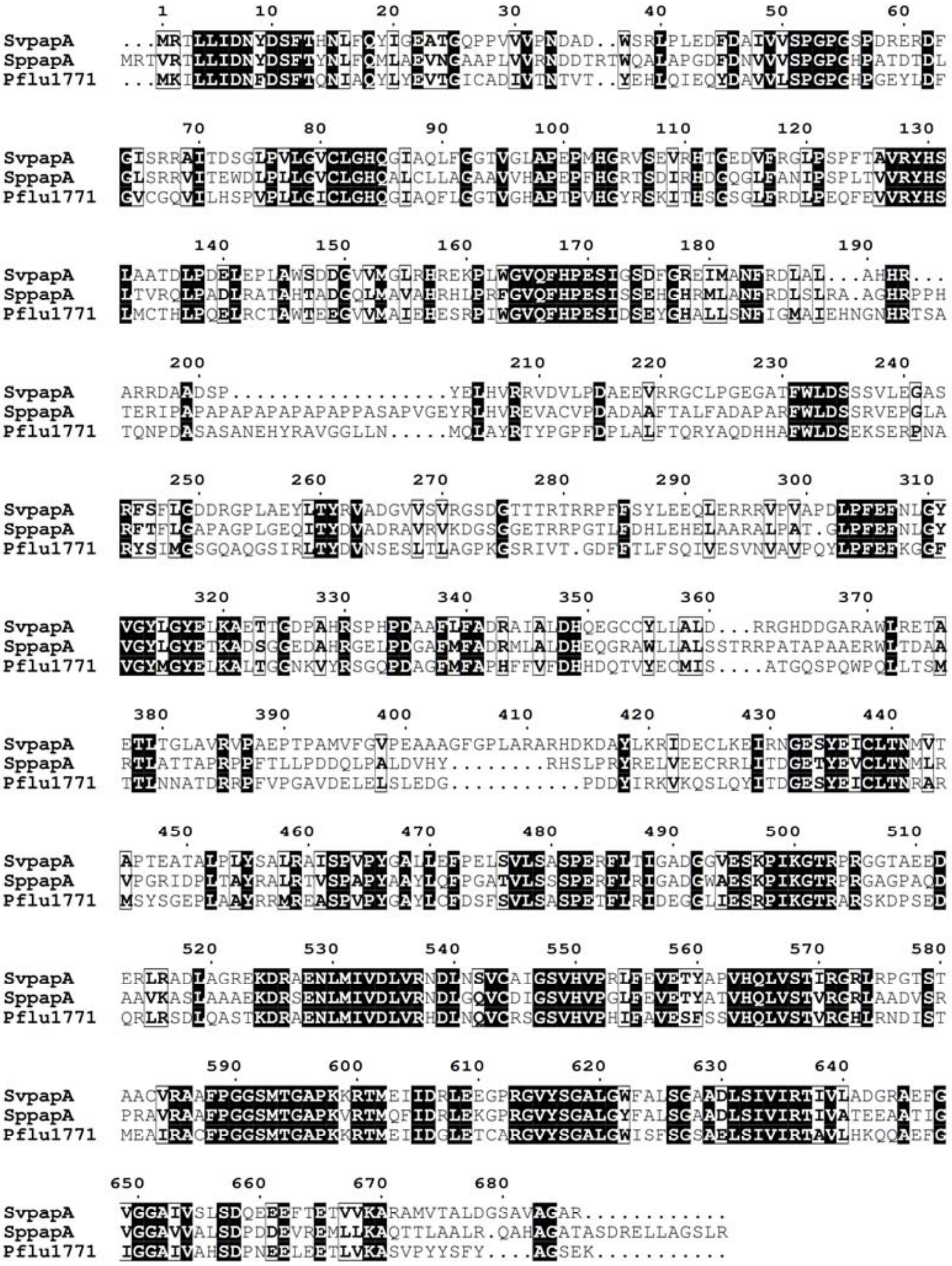

b

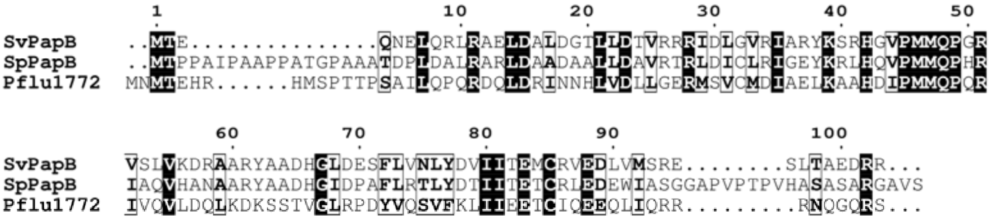

Supplementary Fig. 1 (continued)

c

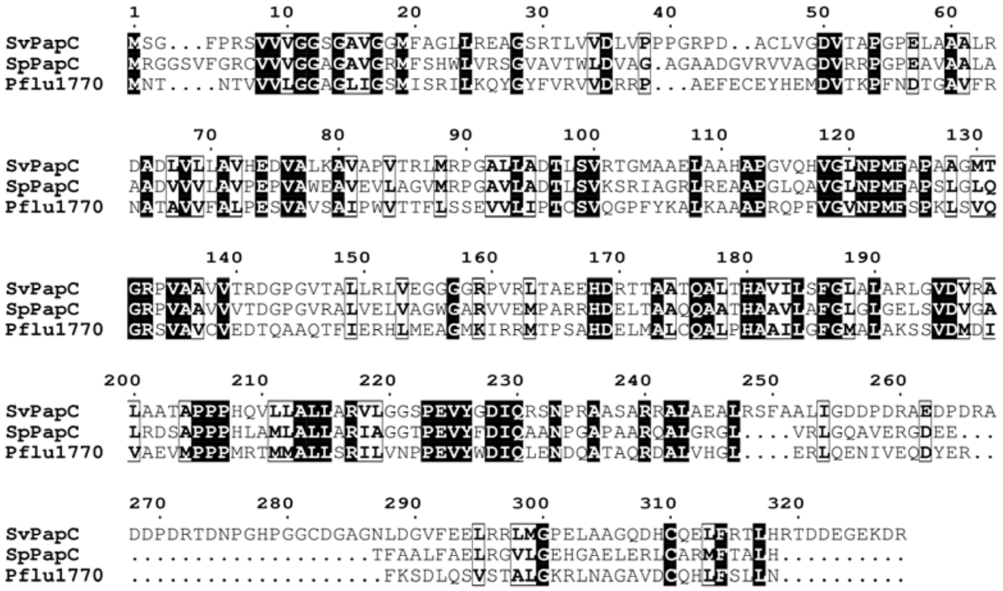

Supplementary Fig. 2

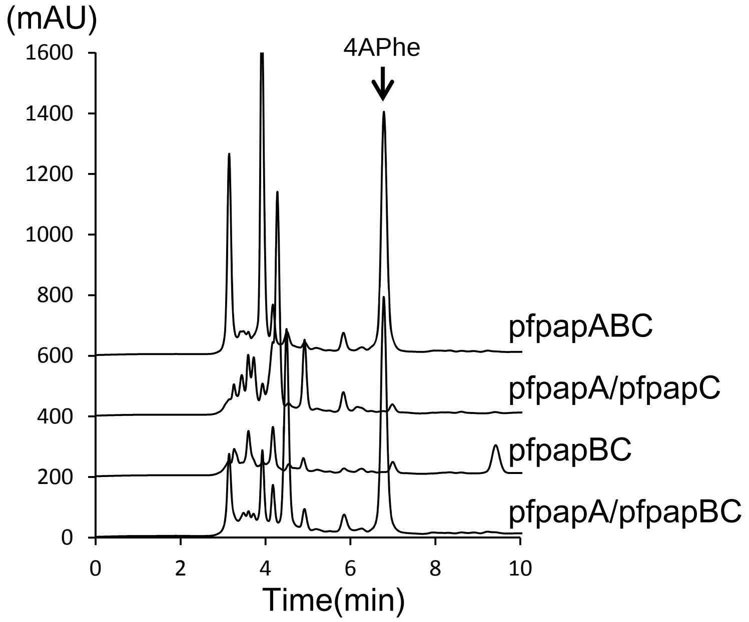

Supplement: Supplementary Information [file srep25764-s1.pdf]
